# Editorial: Personalized therapy in ARDS

**DOI:** 10.3389/fmed.2023.1136708

**Published:** 2023-01-23

**Authors:** Denise Battaglini, Enric Barbeta, Antoni Torres, Patricia R. M. Rocco

**Affiliations:** ^1^Anesthesiology and Intensive Care, San Martino Policlinico Hospital, IRCCS for Oncology and Neurosciences, Genoa, Italy; ^2^Department of Medicine, University of Barcelona, Barcelona, Spain; ^3^Department of Pulmonology, Hospital Clínic of Barcelona, Barcelona, Spain; ^4^Institut d'Investigacions Biomèdiques August Pi I Sunyer (IDIBAPS), Barcelona, Spain; ^5^Centro de Investigación en Red de Enfermedades Respiratorias (CIBERES), Madrid, Spain; ^6^Laboratory of Pulmonary Investigation, Carlos Chagas Filho Institute of Biophysics, Federal University of Rio de Janeiro, Rio de Janeiro, Brazil

**Keywords:** acute respiratory distress syndrome (ARDS), personalized medicine, neurological complications, animal model, mechanical ventilation, COVID-19

## 1. Introduction

In the last decade, research on acute respiratory distress syndrome (ARDS) has made progress in better understanding the variety of presentations, the importance of targeting specific pathophysiological processes, and how these can influence the individual response to treatments within the context of personalized medicine (1). Particularly, the identification of novel biomarkers seems promising in the management of ARDS. In the current Research Topic on “*Personalized Therapies in ARDS*” we encouraged the submission of articles discussing ARDS pathophysiology and classification, pharmacological and supportive therapies, chest imaging, as well as identification of biological sub-phenotypes as a target for potential novel treatment strategies.

One of the main difficulties in experimental studies is to find an effective model which can closely resemble human ARDS (2). Wildi et al. validated an ARDS ovine model in 23 sheep on mechanical ventilation (MV), comparing the mRNA expression of biomarkers in leukocytes, which displayed different sub-phenotypes (hyperinflammatory and hypoinflammatory). The authors found that similar activated pathways might be involved (e.g., oxidative phosphorylation, nuclear factor (NF)-κB pathway) resulting in specific sub-phenotypes, suggesting the importance of omic approaches in ARDS (1).

Superinfections are common in COVID-19 ARDS (C-ARDS) (3). In a prospective multicentric study, Weiss-Tessbach et al. investigated the use of biomarkers in patients with C-ARDS and extracorporeal membrane oxygenation (ECMO). Interleukin (IL)-10 showed the best performance when differentiating secondary fungal/bacterial infections from C-ARDS and/or ECMO associated inflammation.

Another challenge is to classify and identify ARDS severity (4). Hagens et al. classified 401 ARDS patients according to the Berlin definition based on case records using both a dichotomous assessment and an 8-grade confidence scale. Classification was based on confidence grades of three experts through the interclass correlation (ICC). The best ICC was found using an 8-grade confidence scale for lung ultrasound (LUS) (0.49; 95% CI = 0.29–0.63) and computed tomography (CT) scan (0.49; 95% CI = 0.34–0.61). Using the 8-score system instead of dichotomous assessment, the ICC of chest X-ray and CT scan increased by 0.022 and 0.065. Adding information from LUS or chest CT increased ICC by 0.25 when using the 8-grade confidence assessment. This study suggests that ARDS diagnosis requires more than one operator and 8-grade confidence scale.

Even though radiographic assessment of lung edema (RALE) score can be used to evaluate both the extent of edema and ARDS severity (4), its prognostic ability is yet to be determined (5). Worku et al. evaluated the relative changes in RALE score upon venous-venous ECMO initiation in severe ARDS patients. The RALE score demonstrated excellent inter-rater agreement. RALE increased from baseline during the first day of ECMO and this increase was associated with higher baseline APACHE-III scores and greater reductions in tidal volume. This study suggested that an appropriate ventilator setting for patients with ECMO is yet to be defined, and that the RALE score can be considered for the assessment of the optimal MV target to mitigate the risk of atelectrauma.

In contrast with pharmacological therapies, which failed to provide clear beneficial effects in ARDS, some supportive therapies present better results. Nowadays research is focusing on novel supportive strategies, especially MV, to improve patient outcome (6). Soydan et al. performed a cross-over trial in 30 critically ill children comparing automated ventilation with closed–loop control of the fraction of inspired oxygen (FiO_2_) to automated ventilation using manual titrations of the FiO_2_ focusing on time spent in predefined pulse oximetry (SpO_2_) zones. They found that adaptive support ventilation (ASV) using a close-loop control of FiO_2_ titration increased the percentage of time spent within optimal SpO_2_ zones and increased the total number of FiO_2_ changes per patient, suggesting potential advantages with this strategy as compared to manual titration of FiO_2_.

Sandal et al. in a single-blinded randomized cross-over trial, confirmed a better oxygen control using a close-loop oxygen strategy in 23 pediatric patients even when patients were treated with high flow nasal oxygen (HFNO). According to these findings, we can assume that closed-loop oxygen control improves oxygenation in pediatric patients receiving HFNO and ASV for ARDS, thus potentially leading to a more efficient oxygen use, and reduction of the number of manual adjustments.

The need for personalized MV was even more enlightened by a narrative review from Humayun et al.. They confirmed that acute brain injury is a possible consequence of ARDS, thus requiring specific lung protective MV strategies to limit secondary damage to brain. The authors suggest the use of permissive hypercapnia with personalized Carbon dioxide (CO_2_) goals in those without high intracranial pressure. Optimal oxygen targets should be higher than those recommended by guidelines (55–80 mmHg) in case of ARDS and concomitant acute brain injury, while the use of positive end-expiratory pressure around 12 cmH_2_O seems to be safe for the brain. Given the paucity of data in this population, more studies are required to better evaluate MV strategies and blood-gas threshold so as to reduce the risk of brain damage.

Sedatives and anesthetics may impact patients' spontaneous breathing and MV duration. During the pandemic, metanalyses suggested that neuromonitoring patients with C-ARDS can be useful to guide sedation and detect those at risk of neurological complications (7, 8). Tobar et al. in a randomized double-blind trial, found that the use of a multiparameter electroencephalogram (EEG) protocol to guide sedation in C-ARDS as compared to no-EEG monitoring did not increase 30-day ventilator-free days, but reduced propofol administration and deeper sedation 5 days after randomization. These data suggest the importance of investing in future trials testing the impact of multimodal neuromonitoring strategies during sedation and their implications in ARDS patients.

## 2. Conclusions

Our Research Topic on “*Personalized Therapy in ARDS*” highlighted the relevance, current advances, and rationale for personalized approaches in ARDS, including the role of lung imaging, sub-phenotype identification, and need for physiologically based MV individualized approaches in different sub-populations of ARDS patients, including those with acute brain injury.

## Author contributions

DB wrote the manuscript. PR edited the manuscript. EB and AT intellectually contributed and revised the manuscript. All authors approved the submitted version.
